# Cloning, expression and purification of *Pwo* polymerase from *Pyrococcus woesei*

**Published:** 2011-09

**Authors:** Amir Ghasemi, Ali Hatef Salmanian, Nourkhoda Sadeghifard, Amir Ahmad Salarian, Mohammad Khalifeh Gholi

**Affiliations:** 1 Department of Pathobiology, Institute of Public Health, Tehran University of Medical Sciences, Tehran, Iran.; 2 Army University of Medical Sciences, Tehran, Iran.; 3 National Institute of Genetic Engineering and Biotechnology (NIGEB). Shahrak-e-Pajoohesh, 15th Km, Tehran, Karaj Highway, Tehran, Iran.; 4 Clinical Microbiology Research Center, Ilam University of Medical Sciences, Ilam, Iran.

**Keywords:** *Pyrococcus woesei*, cloning, PCR

## Abstract

**Background and objectives:**

*Pyrococcus woesei* is a hyperthermophilic archaea and produces a heat stable polymerase (*Pwo* polymerase) that has proofreading activity.

**Materials and Methods:**

In this study, this microorganism was cultured, its DNA was extracted and the *pwo* gene polymerase was cloned, expressed and purified. The DNA sequence of the cloned gene was verified by sequencing. The *pwo* polymerase gene consists of 2,328 bps (775 amino acids with about 90 kD molecular weight). Cloning was done by GATEWAY™ Cloning System and for purification of recombinant protein; His6x-Tag was added to the C-terminus of the recombinant protein.

**Results and Conclusion:**

We could purify *Pwo* polymerase enzyme by Ni-NTA resin. PCR assay showed that *Pwo* polymerase activity is comparable to a commercial *Pfu* polymerase activity.

## INTRODUCTION

Hyperthermophilic living beings known to date are bacteria which grow at temperatures starting from 80°C and could reach up to 110°C. Some of them are so well adapted to the high temperatures that they do not even grow below 80°C (1–3). These microbes not only tolerate, but grow optimally in habitats normally considered too severe for life. They have adapted to thrive in ecological niches such as deep-sea hydrothermal vents, hot springs, and sulfataric fields (4). Hyperthermophiles occur mainly within the archaebacterial kingdom; some of them are also present within the eubacteria (5). To date, around twenty-two genera of these extreme thermophiles and four unclassified microorganisms have been identified (6). *Pyrococcus woesei*, a hyperthermophile, is a heterotrophic and anaerobic archaeon, with an optimum growth temperature of 98 to 100°C (7). *Pyrococcus woesei* and *P. furiosus* were isolated from marine sediments at the same Vulcano Island beach site and share many morphological and physiological characteristics (8). *Pyrococcus woesei* grows on a variety of a-linked carbohydrates, such as starch and maltose, as well as on -linked glucan substrates, such as cellobiose, laminarin, barley -glucan, and some soluble forms of cellulose (9). The genomic sequences of hyperthermophilic microorganisms are of considerable biotechnological interest because they are natural sources of heat-stable enzymes that can be developed for biotechnological purposes (10). Many interesting enzymes have been isolated from extremophilic microbes (sometimes called extremozymes) (11). *Pwo* Polymerase, Amylase, Pullanase, Glutamin Synthetase I, Galactosidase and Ferredoxin are obtained from *Pyrococcus woesei. Pwo* DNA Polymerase is a thermostable polymerase with an extremely low error rate (18-fold lower than Taq DNA Polymerase) (7, 12–14). Successful cultivation is one of the main problems with these microorganisms. The nutritional needs of these bacteria are complex and involve some unknown, but essential compounds which could be found only in their natural habitat. Here we describe optimized procedures for cultivating *P.woesei*, successful DNA extraction and recombinant production of thermostable DNA polymerase which is commercially important. Also demands for thermostable polymerase with an extremely low error rate are increasing in molecular labs in Iran. Finally, we show the activity of recombinant *Pwo* polymerase in amplification of DNA fragments in a polymerase chain reaction.

## MATERIALS AND METHODS

Standard strain of (DSM 3773) *Pyrococcus woesei* were obtained from (DSMZ, Braunschweig, Germany). This kind of bacteria was routinely grown on batch mode using complex medium supplemented with sea water Marine water and we used Caspian Sea water. The Medium contained KH2PO40.5 g, NiCl2. 6H2O 2.0 mg,Wolfe's Mineral Solution ( see below) 10.0 ml, Yeast Extract (BD 212750) 1.0 g, Bacto Peptone 5.0 g, Sulfur powdered 30.0 g (add separately, see below), Na2S . 9H2O 0.5 g was added just before bacterial inoculation. The salts were added to 1.0 L of sea water. Wolfe's Mineral Solution prepared as follow (g/L): Nitrilotriacetic acid, 1.5 g; MgSO4 . 7H2O, 3.0 g; MnSO4 . H2O, 0.5 g; NaCl, 1.0 g; FeSO4 . 7H2O, 0.1 g; CoCl2 . 6H2O, 0.1 g; CaCl2, 0.1 g; ZnSO4 . 7H2O, 0.1 g; CuSO4 . 5H2O, 0.01 g; AlK(SO4)2 . 12H2O, 0.01 g; H3BO3, 0.01 g; Na2MoO4 . 2H2O, 0.01 g and 1.0 L distilled water. In order to prepare a clear Wolfe's mineral solution it is necessary to dissolve the Nnitrilotriacetic acid in 100 ml water and adjust the pH to 6.5 with KOH. Then remain water (up to 200 ml) was added and the other compound could be added one at a time.

The entire ingredient except sulfur powder and Na2S were added and dissolve completely in seawater. The solution was adjusted to pH 6.4 +/- 0.1 and boiled, cooled and dispensed in 25 ml aliquots into each serum bottle containing the powdered sulfur (0.75 gr). For obtaining an anaerobe condition in each bottle, MART set was used. After three times degassing, the bottles were stoppered with rubber septums and sealed with aluminum crimp seals. The atmosphere above the medium was exchanged with oxygen-free nitrogen pressurization. Prior to bacterial inoculation (1% V/V), the medium was reduced with a sterile neutral solution of sodium sulfide to 0.5 g/L final concentration. Bottles were incubated in100oC for 18h. All chemicals were analytical grade and purchased from Merck (Merck Darmstadt, Germany)

**DNA** E**xtraction from** ***P. woesei***. 25 ml of bacterial culture was centrifuged (5000 rpm 10-15 min., 4°C) and the bacterial pellet was suspended in 2 ml of lysis buffer (100 mM Tris–HCl, 100 ml NaCl,0.5 mM EDTA, pH 8). One milliliter of 10% Sarcosyl (N-lauroylsarcosine, sodium salt) solution, 1 ml 10% sodium dodecyl sulfate and proteinase K (final concentration 1 mg/l) were added for complete cell lysis. After 12h of incubation at 40oC, three phenol–chloroform–isoamyl alcohol (24:24:1) extractions and one additional chloroform extraction were performed. The DNA was precipitated by adding two volumes of cold absolute ethanol and the pellet was washed 3 times with 70% ethanol. The DNA pellet was dried at room temperature and resuspended in TE buffer (10 mM Tris–HCl, 2 mM EDTA, pH7.4) and treated with RNase (5 mg/l) for 1 h at 37°C.

The DNA was electrophoresed on 1% agarose gel and visualized with Ethidium bromide staining and UV transilluminator.

**Primer design and** ***Pwo*** **polymerase gene amplification**. The primers were designed based on published data for *pwo* polymerase gene (Acc. No. D12983) and instruction of Gateway cloning (Invitrogen , USA) kit as follow: Pwo-F-5’ CACCATGATTTTAGATGTGGATTACATA-3’

Pwo-R-5ʹ-GGATTTTTTAATGTTAAGCCAGGAAGT-3>

The Amplification reaction were performed with 100 ng of extracted DNA from *P. woeesei*, 25 mM of each dNTP, 10 pMol of each primers, 2 mM of Mg2+ and 2.5 units of prime STAR® HS DNA polymerase (Takara, Japan). The amplification condition were as follow: one cycle at 95°C for 5 min as initial denaturation, followed by 30 cycles at 95°C/30s, 58°C/30s, 72°C/2.5 min and one cycle at 72°C for 10 min as final extension. The PCR product was analyzed by electrophoresis on 1% agarose gel stained with ethidium bromide.

**Cloning of***** Pwo *****polymerase gene**. Reactions were performed according to the manufacturer's instructions. PCR product was cloned into pENTR/SD/D-TOPO vector (Invitrogen, USA) and then *Pwo* polymerase gene was subcloned to pET301/CT DEST vector (Invitrogen, USA) by a recombination reaction. 5 l of the recombination reaction into (DE3) plysS Competent *E. coli* was transformed. Insertion of *Pwo* polymerase gene in the destination constructs was confirmed by PCR, using primers designed according to the attl sequence located on the pET301/CT DEST vector. Their sequences were attL1 5- G TAC AAA AAA GCA GGC T-3, attL2 5- GTA CAA GAA AGC TGG GT-3. The PCR protocol was designed for PCR colony was like PCR for amplification of *Pwo* Polymerase. The target protein contained 16 additional amino acid residues at the C-terminus, including six histidine residues for purification of the recombinant protein by Ni-NTA resin.

E**xpression and Purification of recombinant** ***Pwo*** **polymerase**. *Escherichia coli* strain BL21(DE3) plysS was transformed with pET301pwo was grown at 37°C in 15ml LB Broth containing 100 mg/ml ampicilin O.N as starter. The starter was added to 500 ml LB Broth containing 100 mg/ml ampicilin to OD 600. IPTG (Fermentas, Lithuani) was then added at the final concentration of 1 mM. The cells were harvested after 4 h by centrifugation and the pellet was stored in -75o for 24h. It was shown previously that *Pwo* polymerase is expressed soluble in cytosol (15). The pellet was heat treated at 75°C for 30 Min after that resuspended in 25 ml of buffer B (50 mM NaH2PO4, 500 mM NaCl, 10 mM Imidazole, pH:8). The cells were disrupted by Lysozyme (4 mg/ml), and the insoluble debris was removed by centrifugation in 13200 rpm for 30 min. The clear lysate (about 20 ml) was then applied directly onto a Ni-NTA Agarose. Ni-NTA agarose 3 times was washed by Buffer C (50 mM NaH2PO4, 500mM NaCl, 20mM Imidazole, pH:8) and 3 times with Buffer D (50 mM NaH2PO4, 500 mM NaCl, 40 mM Imidazole, pH:8). Then recombinant protein was eluted with elution buffer (50 mM NaH2PO4, 500mM NaCl, 1 M Imidazole, pH:8) and the eluted protein was dialyzed against 2 changes of at least 12 hours each of storage buffer (50 mM Tris-HCI pH 7.9, 50 mM KCI, 0.1 mM EDTA, 1 mMDTT, 0.5 mM PMSF, 50% glycerol) at 4°C. After dialysis the resulting protein was diluted 1:1 with sterilized storage buffer and stored at -70oC (16). The Concentration of purified recombinant protein was measured by Bradford method.

**PCR assay**. Activity of enzyme was assayed by PCR reaction by *Brucella* genome as template. *tif* gene (1485 bp) of *Brucella mellitensis* was amplified by His6x-tegged *Pwo* polymerase and commercial *Pfu* polymerase (Bioneer, Korea) as described (17).

## RESULTS

**Culture and storage**. 25ml cultures (1% inoculation) were generally grown for 15 to 18 h at 100o and then transferred to fresh medium or stored at 4o C, which they could be served as inoculums for at least 1 month. After 15 to 18 of incubation at 100o, coccoid organisms became visible in culture. Hydrogen sulfide production and black colony could be two indicators for *P.woesei* growth. Gram staining showed that *P. woesei* is gram positive.

**Fig. 1:**
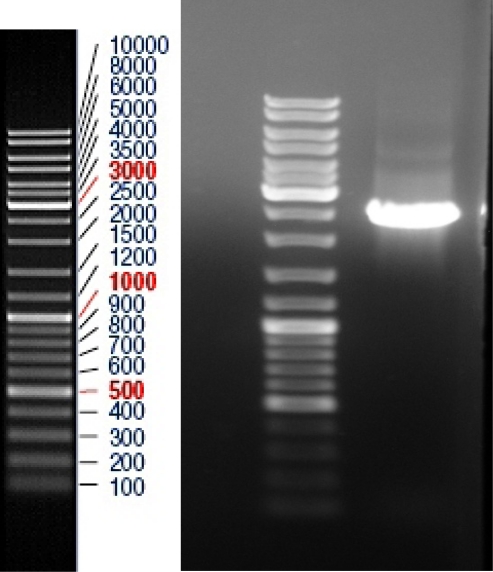
PCR product of *pwo* gene from *pyrococcus woesei*.

**DNA extraction and PCR**. DNA extraction method used in this study could extract *P. woesei* DNA efficiently. The PCR procedure for isolation of *Pwo* polymerase could amplify a single 2325 bp product (Fig. 1). The amplified gene was cloned and sequenced in both directions. The alignment result showed high (> 99%) identity with *Pwo* polymerase gene deposited in GeneBank.

**Construction of the recombinant plasmid producing His6-tagged** ***Pwo*** **Polymerase**. We used the pENTER plasmid containing the attL cassette that can recombine with the pET301/CT DEST plasmid expression vector attB cassette by a LR reaction (18) and allow to transfer of desired gene from pENTER to pET301/CT DEST. The PCR product was inserted into pENTER by TOPO cloning kit (Invitrogen SA). The nucleotide sequences of these constructs were confirmed by sequencing. After that, *Pwo* polymerase gene was transfered to pET 301/CT DEST (Invitrogen, USA) by a recombination reaction and the nucleotide sequence of these construct were confirmed by sequencing analysis too.

The obtained genetic constructs retained the open reading frame and the target protein contained 16 additional amino acid residues at the C-terminus, including six histidine residues for purification of the recombinant protein by Ni-NTA resin.

**Fig. 2:**
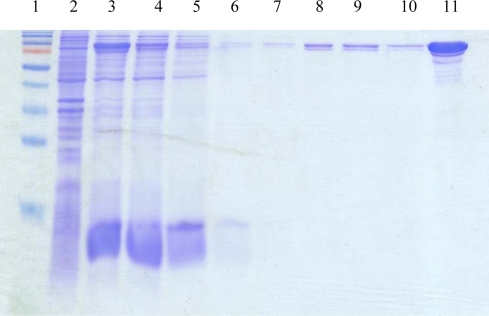
SDS electrophoresis in 12% polyacrylamide gel of the fractions obtained by purification of the recombinant His6-tagged Pwo DNA polymerase.Lane1, molecular weight marker (Fermentase SM 671); Lane 2, Before Induction; Lane 3, After Induction; Lane 4; Flowthrou; Lane 5-7, washed with the buffer C; Lane 8-10, washed with the buffer D; Lane11, eluted recombinant protein with elution buffer.

**Purification of the recombinant His6-tagged** ***Pwo*** **Polymerase and activity assay**. The purity of enzyme was examined by sodium dodecyl sulfate–polyacrylamide gel electrophoresis (SDS–PAGE) (Fig. 2). His6-tagged *Pwo* Polymerase was expressed as a soluble form in the cytosol. Because of heat stable property of *Pwo* Polymerase, heat treating at 70oC was used for denaturing soluble protein but some of *E. coli* proteins still remained soluble after the heating step. The purified recombinant enzyme was obtained by Ni-NTA resin. 14 mg recombinant protein was obtained from one liter liquid culture.

**Fig. 3:**
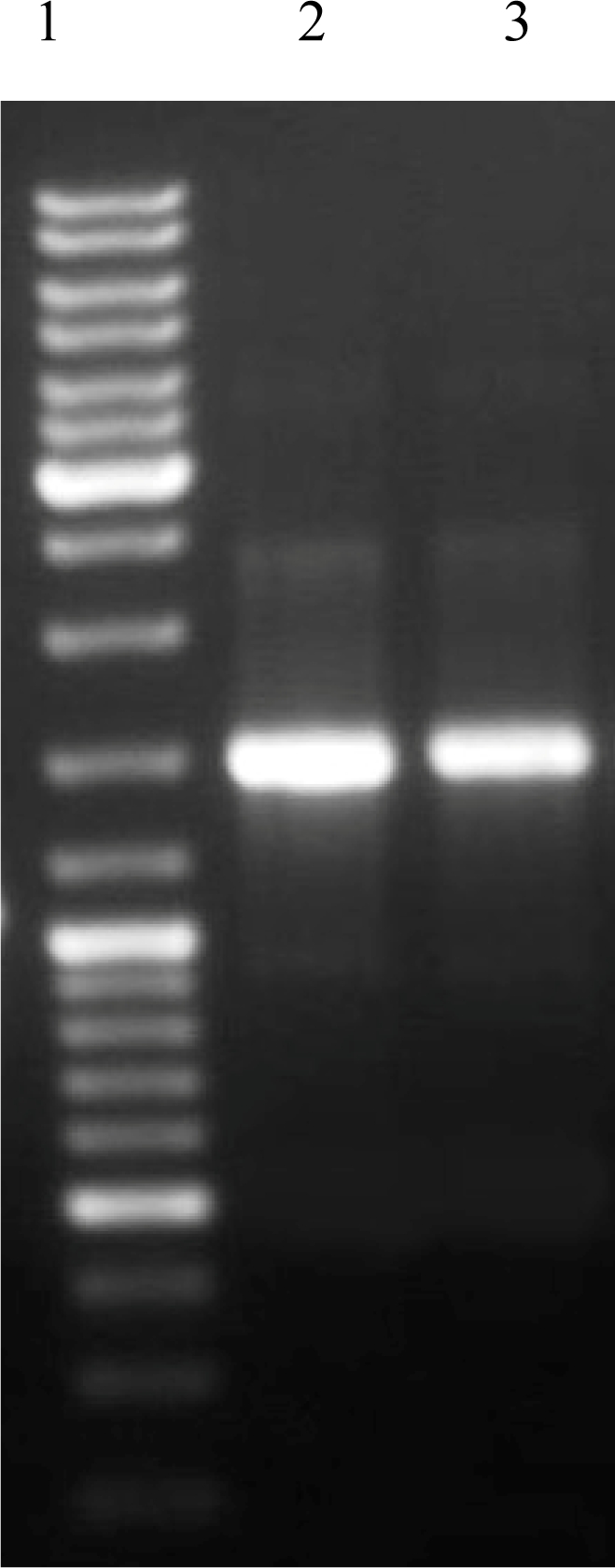
Monitoring of the recombinant His6-tagged *Pwo* DNA polymerase activity.Lane 1, Molecular Marker (Fermentase SM 0333); Lane 2, PCR amplification of *Brucella mellitensis tif* gene by Commercial *Pfu* Polymerase; Lane 3, PCR amplification of *Brucella mellitensis tif* gene by *Pwo* Polymerase.

**Activity of recombinant** ***Pwo*** **polymerase**. PCR activity assay showed that recombinant *Pwo* polymerase has a good activity as compared with commercial *Pfu* polymerase (Fig. 3).

## DISCUSSION

Problem with *P. woesei* cultivation is its complex and udefined requirement for specific elements. To overcome this problem, here we use fresh sea water from Caspian Sea as a source of salt requirements. The methods used for cultivating and DNA extraction of *P. woesei* in this study may help cultivation and DNA extraction of other hyperthermophiles that are in sulphuric hot water sources in some parts of Iran and their probable recognition may introduce new hyperthermophilic strains with special properties.

Due to heat stable proteins incorporating in cell wall structures, the DNA extraction from *Pyrococcus* cell is not as simple as other bacteria (19). So treatment by a powerful proteinase, i.e. Proteinase K, is a key step for breaking the cell wall. The GATEWAY™ Cloning System (Invitrogen, USA) is one of the most powerful systems developed for cloning and expression of recombinant proteins in *E. coli* (20, 21). The pET301/CT DEST vector has a strong T7 promoter and can be grown in combination with pLysS to provide additional stringency (22, 23). The purified recombinant enzymes exhibited high polymerase activity and high thermostability.

The system used in this study for cloning and expression was very efficient and gave 2,500 units/mg of the His6-tagged *Pwo* DNA polymerase activity. This is close to the activity reported for *Pfu* directly purified from *P. furiosus* (31,713 units/mg (24), and from the bacterial pET11 expression system (22,500 units/mg) (25).
